# Neurocalcin-delta: a potential memory-related factor in hippocampus of obese rats induced by high-fat diet

**DOI:** 10.4314/ahs.v17i4.32

**Published:** 2017-12

**Authors:** Wei-Wei Ma, Bing-Jie Ding, Lin-Hong Yuan, Lei Zhao, Huan-Ling Yu, Yuan-di Xi, Rong Xiao

**Affiliations:** 1 School of Public Health, Beijing Key Laboratory of Enviromental Toxicology, Capital Medical University, Beijing 100069, China; 2 Department of Clinical Nutrition Beijing Friendship Hospital, Capital Medical University; 3 Department of Molecular Physiology and Biophysics, Holden Comprehensive Cancer Center, University of Iowa Carver College of Medicine, Iowa City, IA 52242, USA

**Keywords:** Diet-induced obesity, diet-resistant, high fat diet, neurocalcin-delta, proteome

## Abstract

**Introduction:**

Aberrant protein expression within the hippocampus has recently been implicated in the pathogenesis of obesity-induced memory impairment.

**Objectives:**

The objective of the current study was to search for specific memory-related factors in the hippocampus in obese rats.

**Methods:**

Sprague-Dawley (SD) rats were fed either a high-fat (HF) diet or normal-fat (NF) diet for 10 weeks to obtain the control (CON), diet-induced obese rats (DIO) and diet-resistant (DR) rats. D-galactose was injected subcutaneously for 10 weeks to establish model (MOD) rats with learning and memory impairment. After the hippocampus of the rats sampling, the proteome analysis was conducted using two-dimensional get electrophoresis (2-DE) combined with peptide mass fingerprinting (PMF).

**Results:**

We found 15 differential proteins that expressed in the hippocampus in rats induced by HF diet from the 2-DE map. In addition, Neurocalcin-delta (NCALD) was nearly down-regulated in the DR rats compared with CON rats and MOD rats, which was further confirmed by Western blot, real-time PCR and ELISA results.

**Conclusion:**

Our data demonstrates that the differential memory-related proteins were a reflection of the HF diet, but not potential factors in obesity proneness or obesity resistance. Furthermore, NCALD is proved to be a potential hippocampus-memory related factor related to obesity.

## Introduction

Obesity, a chronic metabolic disease, represents one of the most serious public health and societal problems for the coming decades^1^, which affects greater than 35% of the population of U.S and an estimated 670 million people worldwide^2^. Obesity has been associated with a multitude of diseases, including cardiovascular disease, diabetes, hypertension, and certain cancers^3^–^5^. In addition, recent evidence has also highlighted that obesity is associated with cognitive impairments and with an increased risk of developing dementia and alzheimer's disease (AD) later in life^6^–^8^. Epidemiological studies suggest that presence of obesity increases the incidence of numerous neuro-degenerative conditions including AD^8^,^9^. The rate of cognitive dysfunction happened in obese people was significantly higher than that of normal-weighted people^10^–^13^. Such results are essential in understanding the mechanisms for the role of obesity in modulating neuro-degenerative processes^14^. Rodent studies indicate that Sprague-Dawley (SD) rats showed impaired learning and cognitive functions after being fed with the high-fat (HF) diets^15^, complicated with damaged neurons in the hippocampus. This result is consistent with our previous studies^16^,^17^.

Hippocampus-dependent memory appears to be particularly vulnerable to high-fat diets and these deficits can occur rapidly and even prior to weight gain^18^. As the brain cannot synthesize or store energy reserves, foods provide its immediate source of energy to the brain and thereby may influence its structure and functions^19^. Several interactive processes have been proposed to underlie cognitive declines related to HF diets, including oxidative stress and inflammation^20^, increased blood brain barrier permeability^21^,^22^, reduced neurotrophic factors^23^, and insulin insensitivity^24^. Among the pathophysiology of obesity, obesity-induced oxidative stress has been suggested as a potential link between obesity-related metabolic disturbances and chronic diseases. However, the factors related to obesity-induced impairments remain largely unknown. Aberrant protein expression within the hippocampus has recently been implicated in the pathogenesis of obesity-induced memory impairments. Primarily, proteomic techniques are used to examine differences in protein expression in the hippocampus related to obesity. The most traditional and most widely used proteomic method associated with obesity is the two-dimensional polyacrylamide gel electrophoresis (2D-PAGE). In previous researches in obesity, the proteomics assays have been used in the detection of protein expression profiles in serum^25^,^26^, adipocytes^27^, and skeletal muscle^28^. However, there is a paucity of proteomic data on the brain, especially the hippocampus, during obesity. In this study, we reported the evidence of obesity-induced alterations in expression of memory-associated proteins in the hippocampus of obese rats, and thus offered a novel mechanism by which protein expression within the hippocampus is suppressed during obesity.

## Methods

### Animals and diets

All experimental procedures were approved by the Animal Ethics Committee of Capital Medical University and conducted in compliance with the animal-use guidelines. A total of 50 male Sprague-Dawley rats (body weight 140 ∼ 160 g; SPF degree) were purchased from Academy of Military Medical Sciences (Beijing, China). All of the rats were housed in plastic boxes individually at 20 ∼ 23°C with food and water available. Rats were fed with standard laboratory chow for the first week to adapt to the new environment. In the following experimental period, rats were given either a normal-fat (NF) diet (345.3 kcal/100 g, 10% fat content) or a high-fat (HF) diet (435.96 kcal/100 g, 40% fat content). The HF and NF diet formulations (SPF degree) were also purchased form Academy of Military Medical Sciences (Beijing, China).

### Experimental protocol

After the acclimatisation period, 10 rats were randomly assigned to receive a NF diet according to their body weights, and D-galactose (120 mg/kg.d) was injected subcutaneously through the back of the neck for 10 weeks to establish the model (MOD) rats. Another 40 rats were placed on a HF diet for 2 weeks and weighed for body weight. The 10 intermediate weight gainers were then switched back to a NF diet and were designated as controls (CON). The other 30 rats were continually fed with a HF diet continually, and 8 weeks later the upper tertile (n = 10) in gained body weight were designated as diet-induced obese rats (DIO), and the lower tertile (n = 10) in gained body weight were referred to as diet-resistant (DR). Those in the middle tertile (n = 10) were removed from the experiment. The rats were then anesthetized and the blood samples were collected from the heart. The hippocampus of the brain was also all collected for all rats.

### 2-D gel electrophoresis and peptide mass fingerprinting (PMF) by MALDI-TOF-Ms

For 2-DE, equal amounts of the hippocampus tissues of each animal within a group (n=5) were combined to yield the group sample. The hippocampus was weighed and ground into power. The RIPA buffer was added to the powder at 150mg/ml. Then 50 ug/ml RNase and 200 ug/ml DNase were added. Then the solution was placed at 4°C for 15 minutes and centrifuged for 60 minutes at 4°C, 15000 g. The supernates were collected and the protein content was measured with the BCA assay. 10mg/ml of total protein was diluted to 100 µl with rehydration solution (8 M urea, 2 M thiourea, 4% (w/v) CHAPS, 65 mM DTT and 0.5% immobilized pH gradient (IPG) buffer) and applied onto 17-cm, pH3–10 linear IPG strips (Aersham Biosciences, Sweden). The strips were rehydrated for 1 hour at 20°C. The proteins were then focused on the IPGphor system according to the manufacturer's protocol. The strips were equilibrated for 15 min in a solution containing 65 mM DTT, 6 M urea, 20% (w/v) glycerol, 2% (w/v) SDS, and 375 mM Tris-HCl (pH 8.8). The second equilibration step was carried out for 15 min in the same solution except for DTT, which was replaced by 2.5% (w/v) iodoacetamide. Separation of proteins by 2-DE was carried out with 12% SDS-polyacrylamide gel without stacking gel at a constant current of 16 mA/gel for the initial 15 min and 32 mA/gel thereafter until the bromphenol blue dye marker reached the bottom of the gel. Proteins were visualized by coomassie blue staining. The stained gels were scanned and analyzed using an Image Master 2D Platinum 6.0. Protein spots were considered differentially expressed if a significant difference in the normalized spot volume was observed between different groups.

The strips in fresh CCB-stained gel were excised and plated into a 96-well microtitre plate. Excised slices were firstly destained twice with 60 µl of 50 mM NH_4_HCO_3_ and 50% acetonitrile and then dried twice with 60 µl of acetonitrile. Afterwards, the dried pieces of gels were incubated in ice-cold digestion solution (trypsin 12.5 ng/µl and 20mM NH_4_HCO_3_) for 20min and then transferred into a 37°C incubator for digestion overnight. Finally, peptides in the supernatant were collected after extraction twice with 60µl extract solution (5% formic acid in 50% acetonitrile). The peptide solution described above was dried. The 0.8 µl matrix solution (5mg/ml α-cyano-4-hydroxy-cinnamic acid diluted in 0.1% TFA, 50% ACN) was added to dissolve it. Then the mixture was spotted on a MALDI target plate (AB SCIEX). MS analysis of peptide was performed on an AB SCIEX 5800 TOF/TOF. The UV laser was operated at a 400 Hz repetition rate with a wave length of 355 nm. The accelerated voltage was operated at 20 kV, and mass resolution was maximized at 1600 Da. All acquired spectra of samples were processed using the TOF/TOF Explorer TM Software (AB SCIEX) in a default mode. The data were searched by GPS Explorer (V3.6) with the search engine MASCOT (2.3). The searching parameters were as follows: the database for rat, trypsin digestion with one missing cleavage, MS tolerance was set at 100 ppm, MS/MS tolerance of 0.6 Da. The obtained PMFs were used to search through the Swiss-Prot and NCBInr database by the Mascot search engine (http://www.matrixscience.co.uk). Protein with a score greater than 63 were considered to be significant (p<0.05).

### Real-time PCR analysis

The total mRNA from rat brain was purified using the SV Total RNA Isolation system (Beijing ComWin Biotech Co., Ltd., China). The mRNA expression levels of NCALD, were analyzed by real-time PCR. Reverse transcription (RT) was performed with a Reverse Transcription System (Beijing ComWin Biotech Co., Ltd., China). Briefly, double-stranded DNA was synthesized from 1 µg of total RNA and used as a template for the real-time PCR. The sequences of the forward and reverse are shown in Table 2. The real-time PCR reaction system included 0.4 µl of forward primer (10 µM solution), 0.4 µl of reverse primer (10 µM solution), 10 µl of SYBR Green PCR Master Mix, 7.2 µl of nucleus-free water and 2 µl of cDNA, reaching a total volume of 20 µl in each well. Real-time PCR experiments were performed on a CFX Connect Real-Time PCR Detection System as follows: 60 °C for 2 min, an initial denaturation at 95°C for 10 min, and 45 cycles including strand separation at 95°C for 10 s. The housekeeping gene 18S rRNA served as a reference for standardization. All of the measurements were performed in duplicates, and the experiment were repeated once. Fold changes were calculated using the Ct (Ct of the target gene - Ct of the housekeeping gene) method where the fold change is equal to 2^-Ct^ for the gene analysis level.

**Table 2:** Primer sequences and fragment length of NCALD gene

Primer	Sense primer (5′-3′)	Antisense primer (5′-3′	bp
GAPDH	TGGAGTCTACTGGCGTCTT	TGTCATATTTCTCGTGGTTCA	139
NCALD	GCAAATGGAGATGGGACGATAGACT	CTGCCTTGCTGATGTAGCCGTT	138

GAPDH, glyceraldehyde-3-phosphate dehydrogenase; NCALD, Neurocalcin-delta

### Western blot analysis

The hippocampus of the brain in different treatment groups were collected and grinded by tissue grinder in RIPA buffer for 45 minutes at 4 °C, then the homogenized tissues were centrifuged at 15,000 rpm for 20 minutes. The supernatant was separated and collected for protein analysis. The protein concentration was determined by using the BCA protein assay kit (Pierce Biotechnology, USA). Protein samples were loaded and separated by 10% SDS-acrylamide gel electrophoresis and wet transferred to polyvinylidene fluoride blots at the voltage of 60v for 2 hours. The membrane was blocked by fresh blocking buffer (Tris-buffered saline containing 5% skim milk) at room temperature for 1 hour. Immunoblots were performed with appropriate antibodies. Primary antibody (diluted in 1:1000 with TBST and 1% non-fat dry milk) for anti-Neurocalcin-delta (NCALD) (Cell Signaling Biotechnology, USA), was incubated with membrane for 12 hours at 4°C. β-actin (1:1000, Cell Signaling Biotechnology, USA) was used as a housekeeping reference. The proper secondary antibodies were incubated for 1 hour at room temperature. The blots were washed for three times with TBST buffer and protein bands were visualized by using an alkaline phosphatase reaction kit according to the manufacturer's instructions. The FluorChem FC2 software (Alpha Innotech, America) was used to analyze the gray scale value of the protein bands in each group.

### ELISA assay

NCALD protein levels in the hippocampus tissue homogenates of rat brain were measured using the Enzyme-linked Immunosorbent Assay Kits (Enzyme-linked biological technology, Shanghai, China) according to the manufacturer's instruction. Briefly, the tissues were minced in ice-cold PBS (0.01mol/L, pH 7.0–7.2), and then the homogenates were centrifugated for 20 minutes at 15000 rpm. The homogenate (10 µl) and sample diluents (40 ul) were added into the appropriate 96-well plate and incubated for 30 minutes at 37 °C. After washing the 96-well plate for 5 times, the enzyme (50 ul) was added and incubated for 30 minutes at 37°C. After washing the 96-well plate for 5 times again, the color-developing agent A and B were added respectively, and incubated for 10 minutes at 37°C. Finally, the stop solution was also added and the absorption was measured at 450 nm using the microplate reader (Tecan company, Switzerland) immediately. The concentration of NCALD was calculated.

## Statistical analysis

Data were presented as mean ± standard error (S.E) and analyzed with the software SPSS 13.0. The means among groups were compared with one-way ANOVA followed by LSD post-hoc test. A two-tailed p<0.05 was considered to be significantly different.

## Results

### 2-D gel analysis

**Figure 3:**
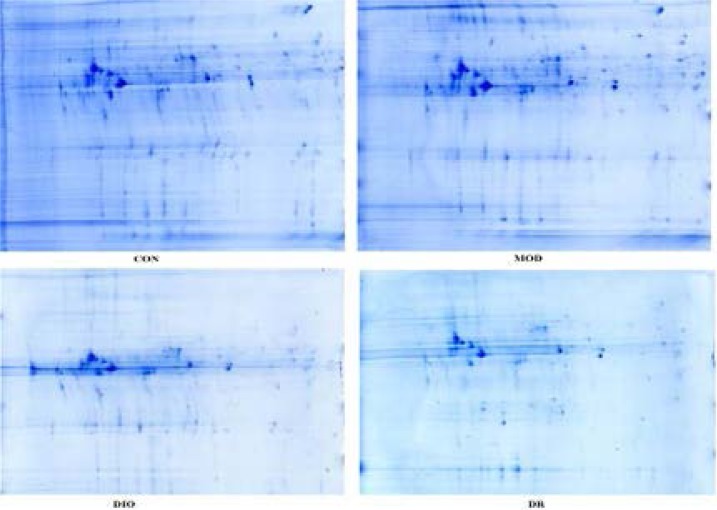
2-DE images of hippocampus proteins of CON, MOD, DIO and DR rats. CON, control; MOD, model rats with learning and memory impairment; DIO, diet-induced obese; DR, diet-resistant.

At the end of week 10, leukemia-associated phosphoprotein p18 (Lap 18) and β-actin were down-regulated in the hippocampus of DIO and DR rats compared with CON rats and MOD rats (all p<0.05). Protein canopy homolog 2 (Cnpy2) and Neurocalcin-delta (NCALD) were down-regulated in DR rats compared with CON and MOD rats, and NCALD was down-regulated in DIO rats compared with CON rat (p<0.05). Heterogeneous nuclear ribonucleo protein K (Hnrnpk) was down-regulated, while ubiquinol-cytochrome c reductase was up-regulated in DIO rats compared with CON and MOD rats (both p<0.05). Neuron-specific calcium-binding protein hippocalcin (Hpca) was down-regulated, while dihydropyrimidinase-related protein 2 (Dpysl2) and gamma-actin (Atcg1) were up-regulated in DR rats compared with CON and MOD rats (all p<0.05). Down-regulated tubulin beta-2A chain (Tubb2a) and up-regulated serum albumin precursor (Alb) were observed in the DR rats compared with other groups of rats (all p<0.05). Tubulin alpha-1B chain (Tuba1b) was up-regulated in DIO rats compared with DR rats (p<0.05). Ubiquitin carboxy-terminal hydrolase L1 (Uchl1) was down-regulated and malate dehydrogenase (Mdh2) was up-regulated in DIO rats compared with MOD rats (both p<0.05). Dismutase was downregulated in both DIO and DR rats compared with CON rats (both p<0.05). The differentially expressed spots are presented in Figure 3 and the identified proteins are listed in Table 1.

**Table 1:** The identified protein in the hippocampus of DIO and DR rat.

Spot No.	Identified Protein	Relative value of the protein expression				Gi accession
		CON	MOD	DIO	DR	
1	leukemia-associated phosphoprotein p18	779.9	649.9	78.5 ab	13.9 ab	134974
2	protein canopy homolog 2	3128.2	987.2 a	373.9 a	13.9 ab	117606182
3	heterogeneous nuclear ribonucleoprotein K	204.7	319.4	9.9 ab	225.5	48429097
4	neurocalcin-delta	1653	1178.4	573 a	128.9 ab	81909955
5	neuron-specific calcium-binding protein hippocalcin	4016.7	4892.9	1997.7	640.1 ab	8850221
6	Tubulin beta-2A chain	5293.1	5797.1	5440.8	1332.1 abc	144587401
7	ubiquinol-cytochrome c reductase	167.4	259.8	1834.4 ab	406.5 a	149045287
8	ubiquitin carboxyl-terminal hydrolase isozyme L1	8.4	399.3 a	31.3 b	67.9	68844977
9	dihydropyrimidinase-related protein 2	1161.7	1298.3	2997.1	3650.5 ab	1351260
10	dismutase	2038.7	1674.3	1002.8 a	204 a	818029
11	malate dehydrogenase	1646.8	614.5 a	3253.4 b	1639.5	81861572
12	beta-actin	4753.3	3317.1	139.9 ab	242.3 ab	46397316
13	gamma-actin	2580.6	3649.9	272.5 b	10864.9 a b	54036665
14	tubulin alpha-1B chain	1509.4	2470.1	4896.3	2293.1^c^	55976173
15	serum albumin precursor	684.7	951.9	1233.8 a	6561.9 abc	158138568

All listed substrates were identified by 2-DE and MALDI-TOF-MS analyses. SWISS-PROT and NCBI protein database were used to evaluate MS data. Relative value was significantly different from those of the CON group:

a *p*<0.05; Relative value were significantly different from those of the MOD group:

b *p*<0.05; Relative value were significantly different from those of the DIO group:

c *p*<0.05; CON, control; MOD, model rats with learning and memory impairment; DIO, diet-induced obese; DR, diet-resistant; NCALD, Neurocalcin-delta.

### NCALD ELISA assay kit

NCALD concentration in the hippocampus of the rat brain is shown in Table 3. DIO and DR rats had significant lower NCALD concentrations compared to the CON and MOD rats (all p<0.05).

**Table 3:** The level of NCALD in brain hippocampus tissue (mean±SE, n=6) of DIO and DR rats induced by high-fat diet

Group	NCALD
	(µg/g prot)
CON	27.06±2.81
MOD	25.74±2.33
DIO	17.97±2.11 a b
DR	10.53±0.77 a b c

Mean values were significantly different from those of the CON group:

a *p*<0.05; Mean values were significantly different from those of the MOD group:

b *p*<0.05; Mean values were significantly different from those of the DIO group:

c *p*<0.05; CON, control; MOD, model rats with learning and memory impairment; DIO, diet-induced obese; DR, diet-resistant; NCALD, Neurocalcin-delta.

### Expression of NCALD in obese rats

**Figure 4:**
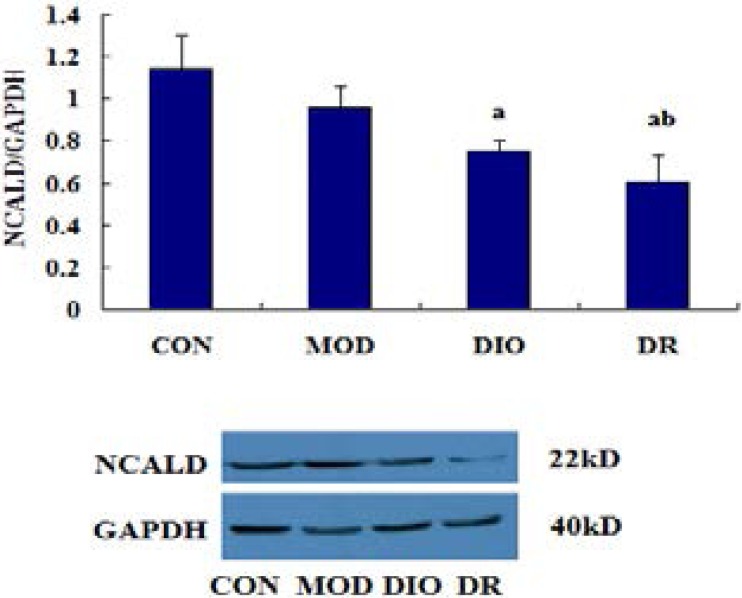
The protein expression of NCALD in DIO and DR rats induced by HF diet. Mean values were significantly different from those of the CON group: ^a^, *p*<0.05; Mean values were significantly different from those of the MOD group: ^b^, *p*<0.05; CON, control; MOD, model rats with learning and memory impairment; DIO, diet-induced obese; DR, diet-resistant; NCALD, Neurocalcin-delta.

**Figure 5:**
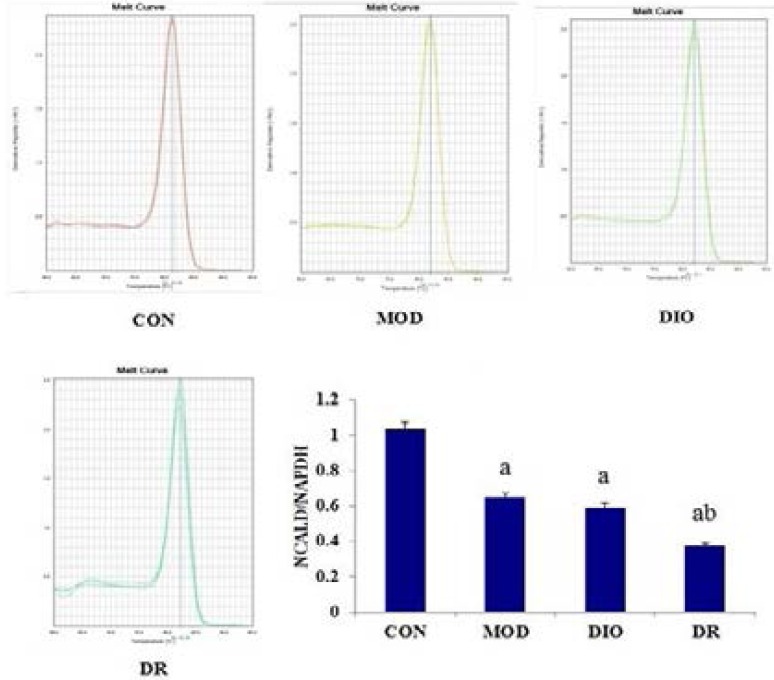
The gene expression of NCALD in DIO and DR rats induced by HF diet. Mean values were significantly different from those of the CON group: ^a^, *p*<0.05; Mean values were significantly different from those of the MOD group: ^b^, *p*<0.05; CON, control; MOD, model rats with learning and memory impairment; DIO, diet-induced obese; DR, diet-resistant; NCALD, Neurocalcin-delta.

As shown in Figure 4 and 5, the WB results showed that the protein expression of NCALD was significantly down-regulated in DIO and DR rats compared with CON and MOD rats (all p<0.05), while the mRNA of NCALD was down-regulated in MOD, DIO and DR rats compared with CON rats (all p<0.05).

## Discussion

In our previous study, rats showed high distinct susceptibility to develop obesity when the rats were fed with a HF diet. In the present study, SD rats were fed with either a HF diet or a NF diet for 10 weeks to generate the CON, DIO and DR rats. The availability of DIO and DR rats are crucial in exploring the mechanisms for development of obesity. D-galactose was injected sub-cutaneously for 10 weeks to establish model (MOD) rats with learning and memory impairments. The final body weight, the perirenal fat, the testicular fat, omental fat and body fat mass of DIO rats were higher than that of DR rats^17^. MOD rats showed impaired learning and memory ability compared with the CON rats^16^. Alerted protein expression within the hippocampus has recently been implicated in the pathogenesis of obesity-induced memory impairments. Thus, in the present study, we aimed to investigate the differential protein expression in the DIO and DR rats by using 2-D gel electrophoresis and PMF.

**Figure 2:**
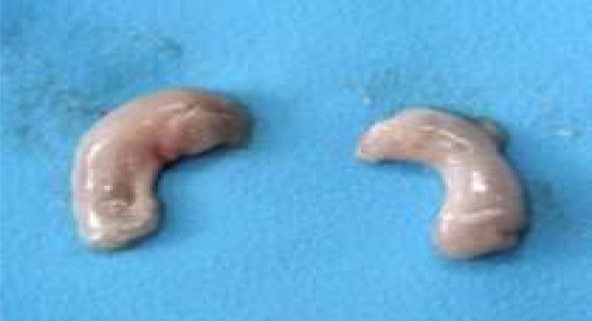
The hippocampus of the rats.

Taken together, the differentially expressed spots are presented in Figure 3 and the identified proteins are listed in Table 1. Our findings suggested that the memory-impairing effects of diet-induced obesity might potentially be mediated by down-regulated NCALD within the hippocampus. NCALD is a member of the neuronal calcium sensor (NCS) family of calcium-binding proteins, which mediates signal transduction in response to calcium in neurons^29^. Furthermore, NCALD may be involved in the processes of spermatogenesis, tumorigenesis and diabetic nephropathy^30^–^32^. In this study, we identified the down-regulated expression of NCALD down regulated in DR rats compared with CON and MOD rats, while NCALD was down-regulated in DIO rats compared with CON rats. Furthermore, NCALD concentration in the hippocampus of the rat brains are shown in Figure 2. DIO and DR rats had the lower NCALD concentrations in the hippocampus compared to the CON and MOD rats and the difference were statistically significant. The results also showed that the protein expression of NCALD was significantly down-regulated in DIO and DR rats compared with CON and MOD rats (p<0.05), while the mRNA level of NCALD was down-regulated in MOD, DIO and DR rats compared with CON rats (p<0.05). These results indicated that NCALD might be a potential memory-related protein in response to HF diets. In addition, the western blot, real-time PCR and ELISA results showed that the NCALD mRNA and protein were more abundantly expressed in the hippocampus of CON and MOD rats but not in the DIO and DR rats, confirming that the HF diet likely contributed to the inactivation of NCALD in the brain, which in turn led to neuronal damages.

Hpca is a protein that buffers intracellular calcium and prevents calcium-induced cell death^33^. A proteomic approach revealed that Hpca expression wasdecreased in vehicle-treated animals with combined middle cerebral artery occlusion^34^, indicating that its decline was likely to be the performance of damage/lesion. In our study, we found the level of Hpca was also down-regulated, strongly indicating reduced Hpca as a principal pathogenic mediator of obesity-induced memory impairments.

β-actin and β-tubulin are essential components of the cytoskeleton and play crucial roles in eukaryotic cells. The actin cytoskeleton functions in the generation and maintenance of cell morphology and polarity, endocytosis, intracellular trafficking, contractility, motility, and cell division^35^.

This result was also consistent with our previous study in the serum proteomics^25^. Ubiquinol-cytochrome c reductase binding protein, a component of the mitochondrial complex III, has been recently implicated in ROS production^36^. High ROS levels are known to have harmful effects on cell growth, resulting in apoptosis. In this study, Ubiquinol-cytochrome c reductase binding protein was up- regulated in the brain of DIO rats, indicating that this protein potentially contributed to the ROS production in the hippocampus of the brain in DIO rats.

Alb protein was down-regulated in DR rats compared with other groups, suggesting their different responses to HF diets. Alb was also found in the brain of AD patients. Thus, the serum albumin expressed in the brain of DIO rats but not DR rats might be a factor leading to obesity and obesity resistance.

Lap18, Cnpy2, Hnrnpk and Uchl1 expression levels in the rat hippocampus might be related to obesity-induced memory impairments, but the reasons for the dysregulation of these genes were still not clear.

## Conclusion

In summary, proteomic analysis of rat hippocampus has provided a useful method to detect differentially expressed proteins in obesity-induced memory impairments. We found the potential memory-related factors as a reflection of HF diet and obesity. Further studies are required to determine the biochemical and physiological functions of these proteins and their relationship with the development of obesity.

## Figures and Tables

**Figure 1:**
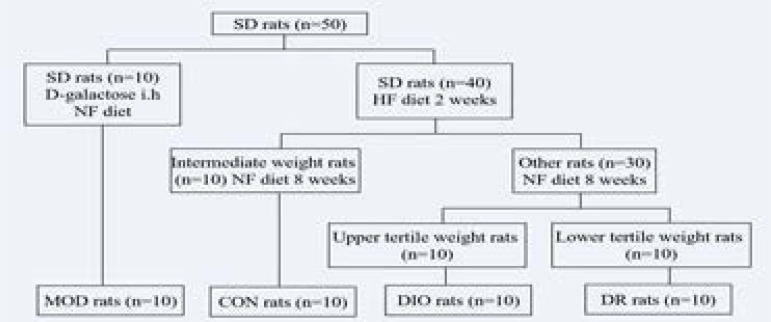
Experimental schedule and groups. CON, control; MOD, model rats with learning and memory impairment; DIO, diet-induced obese; DR, diet-resistant; NCALD, Neurocalcin-delta.
